# Supplementary material for: Promoting Vaccination in India through Videos: The Role of Humor, Collectivistic Appeal and Gender
Source: Vaccines (Basel). 2022 Jul 12;10(7):1110. doi: 10.3390/vaccines10071110 (PMC9323130; doi:10.3390/vaccines10071110)
Supplement: Supplementary file 1 [file vaccines-10-01110-s001.zip › vaccines-1756862-supplementary.pdf]

Promoting Vaccination in India through Videos: The Role of Humor, Collectivistic Appeal, and Gender: Video Repository

For this project eight videos were filmed and shared by adopting a 2 (appeal: individualistic or collectivistic) x 2 (tone: humor or non-humor) x 2 (protagonist gender: male or female) between-subjects design approach to collect data. Below are the corresponding YouTube links to the eight videos created for this project.

Table S1. Video Repository

**Video Content**	**YouTube Link**
Female Collective Serious	https://youtu.be/hqMPFO0pEP0 accessed on 30 April 2022
Female Individual Serious	https://youtu.be/9xyEL4otrhE accessed on 30 April 2022
Female Collective Comic	https://youtu.be/AHiikvtkHVk accessed on 30 April 2022
Female Individual Comic	https://youtu.be/YOOSWHH4gmU accessed on 30 April 2022
Male Collective Serious	https://youtu.be/V8lYT14XiiA accessed on 30 April 2022
Male Individual Serious	https://youtu.be/Ldy2ZuVsyKs accessed on 30 April 2022
Male Collective Comic	https://youtu.be/2zFZM9NjioU accessed on 30 April 2022
Male Individual Comic	https://youtu.be/UO8350oa6l0 accessed on 30 April 2022
